# Plant-Based Meat Substitutes in the Flexitarian Age: An Audit of Products on Supermarket Shelves

**DOI:** 10.3390/nu11112603

**Published:** 2019-10-30

**Authors:** Felicity Curtain, Sara Grafenauer

**Affiliations:** 1 Grains & Legumes Nutrition Council, Mount Street, North Sydney 2060, Australia; sarag@glnc.org.au; 2 School of Medicine, University of Wollongong, Northfields Avenue, Wollongong 2522, Australia

**Keywords:** meat substitute, meat alternative, plant protein, sustainability, flexitarian, vegetarian, vegan

## Abstract

Demand for plant-based meat substitutes is growing globally for nutritional and environmental reasons, with Australia the third-fastest growing vegan market worldwide. This study aimed to profile and compare plant-based meat substitutes (mimicking meat) with equivalent meat products, and 2015 data. An audit undertaken in May (updated in September 2019) from four metropolitan Sydney supermarkets (Coles, Woolworths, Aldi, IGA), collected nutrition information and Health Star Rating (HSR) from 137 products (50 burgers, 10 mince, 29 sausages, 24 chicken, 9 seafood, 15 other). Mean (± standard deviation (SD)) and median (range) was calculated for nutrients and HSR. Plant-based options were generally lower in kilojoules, total and saturated fat, higher in carbohydrate, sugars, and dietary fibre compared with meat. Only 4% of products were low in sodium (58–1200 mg/100 g). Less than a quarter of products (24%) were fortified with vitamin B12, 20% with iron, and 18% with zinc. HSR featured on 46% (3.6–4.4 stars). On-pack claims were vegetarian/vegan/plant-based (80%), protein (63%), non-genetically modified/organic (34%), gluten free (28%). Product numbers increased five-fold (↑429%) in four years. The plant protein trend has prompted innovation in meat substitutes, however wide nutrient ranges and higher sodium levels highlights the importance of nutrition guidelines in their development to ensure equivalence with animal-based proteins.

## 1. Introduction

Food choice is strongly linked with human health and the health of our environment, with excessive meat consumption often viewed as detrimental for both factors [1]. Globally, agriculture and food production is responsible for more than 25% of all greenhouse gas (GHG) emissions [1], with effects widely varied based on food-type. It is well-accepted that animal-based foods have a greater environmental impact that plant-based foods, emitting more GHG, requiring more land and nitrogen, and impacting terrestrial and aquatic biodiversity [2]. Ruminant meats (beef and lamb) are considered of greater consequence than eggs, seafood, poultry, and pork [3] and global meat consumption has increased 58% over the past 20 years to 2018. Consumption, now at 360 million tonnes is accounted for through population growth (54%), and the remainder is due to increased consumption, which is expected to increase further [4,5].

Suboptimal diets are responsible for more deaths than any other risk factors globally, including tobacco smoking, causing an estimated 11 million deaths and 255 million disability-adjusted life years [6]. Worldwide, intake of red meat surpasses what is considered optimal by 18% (led by Australasia, southern, and tropical Latin America), and consumption of processed meat is 90% greater than the optimal amount (led by North America, Asia Pacific, and western Europe) [6]. However, compared to other dietary risks, high intake of red and processed meat ranked at the bottom end (at number 13, and 15 respectively) for death and disability. Conversely, high intakes of sodium, and low intakes of whole grains, fruit, vegetables, nuts and seeds all featured in the top five risk factors [6]. Nonetheless, observational research has associated high intakes of red and processed meats with an increased risk of colorectal cancer [7,8], all-cause mortality [9], and cardiovascular disease [10,11], with Australia’s National Heart Foundation recently endorsing a weekly limit of 350 g red meat [11], lower than the current Australian Dietary Guidelines limit of 455 g red meat/week [12].

Despite this, Australians consume an estimated 115 kg meat per capita (including beef, lamb, mutton, chicken, pork and fish), equating to 2.2 kg per week [4], greatly exceeding recommendations. Increasing consumption of plant-based foods, and decreasing animal foods, has been described as a health and environmental ‘win-win’ by authors of the EAT Lancet report, which describes targets for a healthy diet and sustainable food production system [13]. Greater knowledge and awareness means sustainability has emerged as a significant focus for food producers and consumers alike, based on three dimensions; ecological, social and economic, and now a fourth dimension focused on health [14].

Globally, dietary guidelines promote protein-based foods in various ways. A recent comparison of 90 guidelines worldwide found one third refer solely to animal sources of protein (such as meat, poultry, fish, eggs, and sometimes dairy foods), while 37% include plant-based protein sources in the same group, such as legumes, nuts and seeds [15]. This is the case with the Australian Dietary Guidelines, which are based around five core food groups, one being ‘lean meat and poultry, fish, eggs, tofu, nuts and seeds and legumes/beans’ [12]. Similarly, the newly revised Canadian Dietary Guidelines specifically emphasise a shift towards a more ‘plant-based’ diet, encouraging foods like legumes, nuts, seeds, tofu, and fortified soy beverages, before mentioning animal-based foods such as fish, shellfish, eggs, poultry, lean red meat and dairy foods [16].

The focus on plant-based proteins has created a significant opportunity within food industry against a backdrop of health and environmental concerns. Meat substitutes such as tofu and textured soy protein products have existed in the western world since at least the 1960s [17,18], however it now appears that the target has moved from niche, to more mainstream, with products designed specifically to appeal to meat-eaters. Products resembling burger patties, mince, sausages, and chicken are displayed alongside meat in the chilled cabinets and many mimic meat products directly, with ‘bleeding’ burgers and other products designed to exhibit ‘meaty’ characteristics [19]. Since 2015, launches of plant-based meat substitutes that mimic the taste, texture, and appearance of animal-based products have exceeded 4400 products worldwide [20]. Modelling suggests the plant-based meat market in Australia alone may reach $3 billion in sales by 2030 [21], however it is unknown if consumers are truly viewing these as a direct substitute for meat, with little consumer research [14], and a poor understanding of how these products compare with currently available meat cuts and traditional forms of meat such as burgers, mince and sausages.

The more recent growth in the plant-based meat segment is aligned with the interest in plant-based diets as a healthier dietary pattern. Australia is considered the third-fastest growing vegan market worldwide (behind the United Arab Emirates and China) [22]. The number of adults following vegetarian or almost vegetarian diets increased from 1.7 million to 2.1 million (11.2% of the total population) from 2012 to 2016 [23], a number that is anticipated to increase in alignment with global research findings. Alongside this is the trend towards eating less meat or a “flexitarian” diet approach, also being adopted by more Australians to improve nutrient density and reduce their environmental impact, although they may still consume meat occasionally [19].

However, the absence of universal regulations around the naming of meat substitutes creates an ethical dilemma, and it has been suggested that plant-based meats may mislead consumers into assuming their nutritional profile mirrors animal-based meat [24]. This has become a global political issue, with lobbying in Australia, the USA, and the European Union underway to ban manufacturers from insinuating plant-based meats are in fact ‘meat’, by using meat-related words and imagery [25,26]. Without standards for manufacturers to adhere to, there is limited understanding of whether products provide the range of nutrients naturally found in meat, particularly adequate iron, vitamin B12, and zinc, all of which are naturally present in meat [27]. Equally, while traditional meat products fall within recently proposed voluntary reformulation targets suggested as part of the Australian Healthy Food Partnership, vegetarian/vegan products are specifically excluded, further limiting regulation around their nutritional composition [28].

In light of growing consumer interest in alternatives to traditional animal proteins, this study aimed to provide an overview of currently available plant-based meat substitutes available on Australian supermarket shelves, nutrition composition compared to animal products of comparable culinary use (burgers, sausages, and mince) and changes in the make-up of the category from 2015 data.

## 2. Materials and Methods

A recognised process was used to conduct an audit of plant-based meat substitutes [29,30] in the four major supermarkets (Aldi, Coles, IGA, and Woolworths) of metropolitan Sydney in June 2019, replicating a process that was conducted on the same category in 2015. These supermarket chains represent more than 80% of total Australian market share [31], and were chosen in an effort to reflect choices available to the majority of Australian shoppers. Researchers used smartphones to capture all data on food packaging, including ingredients, nutrition information panel (NIP), health and nutrition claims, country of origin, Health Star Rating (HSR), and any additional logos and endorsements.

Products accounted for in the audit included those designed to mimic meat, yet made from plant-based ingredients. Following data collection, products meeting this criteria were grouped into common categories based on their similarity to meat-based products and dishes, including plant-based burgers, sausages, mince, chicken, seafood, and an additional ‘other’ category with products that fell outside of these categories (Table 1). Products excluded were vegetarian foods not specifically created to imitate meat products, such as tofu, tempeh, and falafel. A supplementary internet search was conducted through supermarket websites and identified manufacturer websites using key words such as ‘meat alternatives’, ‘meat substitutes’, ‘meat-free’, ‘plant-based’, ‘vegan’, and ‘vegetarian’, to ensure all available products were captured. This process was repeated in September 2019 to capture additional products that appeared on supermarket shelves after the initial data collection phase.

Data from photographs was then transcribed into a Microsoft Excel spreadsheet (Redmond, WA, USA) for analysis. Information for the data entry included the NIP per serve and per 100 g, ingredients, HSR, percentage of whole grains and legumes, nutrition and health related claims, including protein, dietary fibre, saturated fat, and sodium. Eligibility for products to make nutrition content claims was assessed in line with Food Standards Australia New Zealand (FSANZ) [32] and the Grains and Legumes Nutrition Council Code of Practice for Whole Grain Ingredient Content Claims (The Code) [33], and HSR was calculated for all products that did not display the system on-pack, using the HSR website calculator [34]. A second, independent reviewer checked data for any inconsistencies and errors, and the number and type of products collected were compared with 2015 data that followed the same process, to assess changes in numbers and types of products.

In order to compare meat substitutes to their equivalent animal-based versions, nutrition composition data was obtained for mince and sausages through FSANZ’s Australian Food Composition Database [35]. Information for burgers did not exist within this database, so nutrition data was averaged from ten products found on Coles [36] and Woolworths [37] websites. For all meat products, an estimated HSR was calculated using the HSR website calculator [34]. As products within the chicken, seafood, and ‘other’ categories varied widely in their animal-based comparisons, these were excluded from this comparison with animal meat products. Where relevant, average nutrition content of meat substitutes were compared with corresponding voluntary sodium and/or saturated fat reformulation targets for ‘crumbed and battered proteins’, ‘processed meat’, ‘sausages’, ‘bacon’, ‘ham’, and ‘processed meats’, proposed by the Healthy Food Partnership [28].

### Statistics

All data were checked for normality using the Shapiro–Wilk test (IBM SPSS^®^, version 25.0, IBM Corp., Chicago, IL, USA) and mean and standard deviation were presented in addition to median and range as only energy, protein, fat and dietary fibre were normally distributed. As expected, there were missing values for dietary fibre, sugars, sodium and iron as these would not be presented in the NIP unless specifically added to the products, therefore these nutrients were analysed separately.

Independent sample *t*-tests (IBM SPSS^®^, version 25.0, IBM Corp., Chicago, IL, USA) were used to compare differences in nutrients per 100g between meat and plant-based meat substitutes for the burger, sausage, and mince categories with data sourced from FSANZ or online supermarkets as described above.

## 3. Results

Data from 137 plant-based meat substitute products were collected, including 50 burgers, 29 sausages, 10 mince, 24 chicken, 9 seafood, and 15 ‘other’ meat substitutes (including ‘vegie roasts’, deli slices such as mock ham and bacon, and tinned nut meat). The number of products overall had increased more than five-fold compared to 2015, with greatest growth seen in burgers (+614%), and seafood emerging as a new category, with no products previously captured from this group (Table 2).

When considering country of origin, the majority of products (61%) were made in Australia. This was followed by 12% from South Africa, 9% from United Kingdom, and 7% from New Zealand, with USA, Canada, Thailand, Taiwan, and Denmark producing smaller numbers of products.

### 3.1. Nutrients and Health Star Rating

Table 3 outlines the key nutrients reported on packs as mean (±SD) and median (range) per 100 g for each of the six categories examined, as not all nutrients were normally distributed. If not reported, the HSR was calculated and the mean is reported.

Mean energy ranged from 574–830 kJ/100 g with protein contributing 9.6–14.5 g/100 g across the categories. Fat was 5.4–9.4 g/100 g with plant-based chicken at the highest end of this range and saturated fat, 1.4–2.4 g/100 g where plant-based sausages were highest. Carbohydrate ranged from 7.9–16.7 g/100 g and dietary fibre was 3.6–5.9 g/100 g with the greatest amount found in burgers and mince. Although mean sodium was generally less than 500 mg/100 g (372–568 mg/100 g), there was a wide range, with products containing up to 1200 mg/100 g or 3 g of salt. Median iron was similar across all products (3–3.9 mg/100 g) except in seafood where the iron was not declared. HSR featured on 46% of products and, when considering all scores (both on-pack, and calculated if not reported), a relatively high score of between 3.6–4.4 (out of a possible 5) stars was determined.

### 3.2. Comparison with Animal Meats

As consumers may directly replace animal meat products with plant-based meats, a comparison in nutrients and HSR between burgers, mince and sausages was conducted and presented in Table 4.

Plant-based meats tended to be lower in kilojoules than animal meat varieties, reaching significance in sausages and mince. A similar difference was seen in total fat and saturated fat, but was significant only for burgers and sausages, in favour of plant-based products. As expected, plant-based products were higher in carbohydrates, sugars and dietary fibre. Sodium in plant-based mince was approximately six times higher than the sodium of meat mince, however the reverse was true for sausages, where meat sausages contained 66% more than the plant-based sausages. There was no difference in iron content of mince or sausages, while iron content was not reported in meat burgers, so no comparison was possible with plant-based burgers.

### 3.3. Ingredients

As plant-based meats are a manufactured product, Table 5 outlines the potential ingredients linked with the key nutrients in these foods (although there was cross over between some food sources and nutrients). It is important to note that less than a quarter of products (24%) were fortified with vitamin B12, 20% of products were fortified with iron, and 18% with zinc.

As shown in Table 5, grains and legumes were common ingredients in meat substitutes. Whole grains such as brown rice, buckwheat, sorghum, quinoa, and millet were found in 8% (*n* = 11) of all products, ten of these being burgers (20% of the burger category), and one falling into the ‘other’ category. Interestingly, six products were eligible to make a whole grain content claim in line with The Code (≥8 g whole grain per serve), and these provided an average of 28g whole grain per serve, meeting 58% of the 48g daily target intake for whole grain [38]. More than two-thirds of all products (35%, *n* = 48) included legume ingredients, such as adzuki and black beans, chickpeas, and lentils, with products containing between 9%–65% legume ingredients.

### 3.4. Comparison with Proposed Reformulation Targets for Equivalent Animal Meats

Although vegetarian/vegan meat substitutes were excluded from the Healthy Food Partnership proposed reformulation targets for meat products, equivalent plant-based products were compared to relevant targets, and the proportion meeting these were determined (Table A1 in Appendix A). Relevant targets included animal meat-based sausages, crumbed/battered meat, poultry, and seafood, bacon, ham, and processed meats. With the exception of sausages, which has a target for both sodium, and saturated fat, the remaining five categories only prescribe a target for sodium. From a theoretical point of view, the majority (90%) of plant-based crumbed/battered meat/poultry were compliant with the proposed sodium target (710 mg/100 g), just over half (55%) of plant-based sausages were compliant (540 mg/100 g), and only 20% of crumbed seafood (270 mg/100 g). Conversely, almost all plant-based sausages (96%) were in line with the proposed target for saturated fat content (7 g/100 g). There were three plant-based ‘other’ products for comparison, one in each of the bacon, ham, and processed meats categories. Only the plant-based ham product was compliant with the proposed target (1005 mg/100 g), while the bacon product (1005 mg/100 g) and processed meat product (720 mg/100 g) both exceeded the sodium target.

### 3.5. Packaging Claims

Table 6 outlines the most common on-pack claims, the top claims being vegetarian, vegan, plant-based, or meat-free, featured on more than 80% of all products. Just over 60% of all products made a nutrition content claim regarding protein, such as ‘high in protein’, or specifically referring to ‘plant-based protein’, compared to the 77% that were eligible to do so, with ≥5 g per serve. Although 58% of products were eligible to make a dietary fibre claim (with ≥2 g per serve), only 39% included this on-pack. Claims assuring the absence of genetically-modified ingredients were common, on 35% of products, along with dairy-free and gluten-free claims, featured on 26% of products. In line with the percentage of fortified products (Table 5), only around one in five products made a claim around minerals (such as iron or zinc) and vitamins (such as vitamin B12). Although 20% of products were considered low in fat, only 15% claimed this on-pack, and only 4% were eligible to claim low in sodium, with ≤120 mg per 100 g but no products made this claim.

## 4. Discussion

Meat consumption is growing globally [5,39] alongside recommendations to consume protein from plant sources. Although plant-based foods (both natural and manufactured) have been available for many years, the rapid and recent rise in the availability of new plant-based meat substitutes means that they have not been specifically included or addressed in country-specific guidelines [15] or in documents such as EAT Lancet [13]. Yet, it has been said that moving to a diet based on more whole and plant-based foods could be ‘one of the most important dietary strategies at a global level both for the planet and for human health’ [3]. The high level of community concern in Australia and globally regarding the environmental impact of the food supply may mean that individuals are more highly motivated by their individual environmental impact compared with health concerns when making food choices [3,40]. As a consequence of these factors, industry is anticipating growth in this category, which generated approximately $150 million dollars in Australian retail sales in 2018–2019, with suggestions based on a modest growth trajectory that the segment could reach between $1.4 to $4.6 billion in retail sales by 2030 [21]. Understanding the nutrition implications and the profile of this category of products is important, as more people adopt flexitarian, vegetarian and vegan dietary patterns. Also, there appears to be a ‘health halo’ effect [41] surrounding plant-based meat substitutes that leads to a healthier perception, which may not be entirely justifiable. This research points to some limitations in the formulations of products, with some being higher in sodium, a finding supported by a recent report from The George Institute for Global Health, which reported similar sodium levels in plant-based burgers and sausages to the current study [42]. Importantly, many products fell short in terms of equivalence to similar meat varieties particularly in respect to micronutrients such as iron, zinc and Vitamin B12. It was beyond the scope of this research to highlight limitations in other micronutrients such as selenium, phosphorus, niacin or in terms of the precise amino acid profile, but these would potentially also be limitations for plant-based meat substitutes [10].

According to a recent review of consumer research, Weinrich found that environmental arguments were not the basis for decisions to purchase and consume from this category of plant-based meat substitutes. Taste, appearance and availability were far more important [14]. The number of products available in mainstream supermarkets is five times that of 2015 and now products are often placed in the chilled meat section adjacent to meat, many with a clear window as part of the packaging so the appearance and similarity to meat is in view. Nath (2011) explored reasons for consuming plant-based meat substitutes in an Australian population and found that plant-based meat substitutes were a valuable aid in converting to a meat-free diet (in this instance for moral and health reasons) [43]. They found the products provided a social facilitator, allowing consumers with preferences for plant protein to engage in the social aspects of eating particularly at family dinners, where similar meat-based products would be consumed, and at other special festivities [43]. Overall, they were seen as a convenient and easy option to prepare in replacement of commonly consumed meals. Interestingly, these authors also found that consumers who identified as vegetarians and vegans questioned the logic of consuming foods that resembled meat and this sentiment has been echoed by other researchers [17,43].

While this research was focused only on supermarket based products, two known Australian fast food chains are also producing products utilizing plant-based meat substitutes. At the fast-food chain Grill’d, the Beyond Burger^TM^ is used in five meat-free options [44] and through leveraging the established Whopper^®^ brand at Hungry Jacks^®^ a Vegan Cheeseburger is available [45] with more products expected to enter the market via other large chains in the near future. It is likely that the opportunity to purchase via a fast food outlet will help to grow consumer acceptance. With a lower level of commitment attached to a fast-food purchase, this may be a convenient entry-point for many to this market, with taste as the key driver rather than the ‘health halo’ as may be the case with purchases from the supermarket. As the Beyond Burger^TM^ brand is also available in the supermarket (and was captured in this audit), this may further encourage purchasers to recreate these meals at home.

With the anticipated growth in the plant-based meat substitutes category, it may be pertinent to suggest that reformulation targets (for example, the Healthy Food Partnership reformulation targets in Australia), are also considered for this category as a way of future-proofing the food supply. Nutritionally, the plant-based meat substitutes segment could be compared with plant-based milk alternatives, those made from almonds, oats, soybean and coconut. Unless fortified, these milks are not equivalent to traditional cow’s milk, as they are not naturally rich in nutrients like protein and calcium [46,47]. However, increased popularity and consumption of plant-based milk alternatives (which now make up 7% of all milk consumed in Australia [48]), has led to greater recognition at a regulatory level. For example, The Healthy Food Partnership has set draft sugar reformulation targets for flavoured milk alternatives, in addition to dairy milks [49], in stark opposition to plant-based meat substitutes, which were excluded. Results from this study show a similar stance is needed for plant-based meat substitutes, as a hypothetical comparison between these and meat products showed varying results—some were compliant, but other categories had 50% of products that were non-compliant with sodium targets despite being an entirely manufactured food. Public Health England included meat alternatives in their salt-reduction targets for 2017; with ‘plain meat alternatives’ (tofu, meat-free mince, plain pieces and fillets) at 250 mg sodium, other meat-free products (sausages, burgers, bites, falafel) at 360 mg, and meat-free bacon at 750 mg [50]. Comparing products in the current study, only one of the 10 ‘plain meat alternatives’ and 38% of other meat-free products were compliant, while the one meat-free bacon product exceeded the target, highlighting the need for industry guidance. Equally, fortification is key to consider. As the category continues to grow in the number of products, and consumer acceptance follows, it will become more important to ensure that regulations are in place aiming for a nutritional equivalence to meat to prevent issues with low levels of iron and vitamin B12 intake, and potentially anaemia in vulnerable segments of the population.

In a study from the Netherlands, Schösler (2012) found that the most common foods chosen to substitute for meat (in ascending order) were fish and eggs, followed by cheese, with plant-based meat substitutes ranked further down the list [51]. Lentils, pulses, nuts ranked even lower on the preference list, with the author stating that lentils and other pulses are a challenging pathway as they disrupt familiar meal formats and it requires active effort to break from existing conventions where meat provides the structural aspect to the meal. Other options, such as seitan, tempeh, tofu ranked last [51]. Legumes were found in more than two-thirds of products captured within this audit; an important finding as modelling shows Australians need to increase current levels of legume intake by 470% to meet recommended amounts [52]. Given the known barriers to legume consumption, such as a lack of knowledge in how to prepare them, and time constraints [53], plant-based meat substitutes may offer a convenient and surreptitious way to increase intake, a conclusion supported by others. [53,54]. Similarly, in this study, 20% of burgers contained >8 g/manufacturer serve of whole grain, presenting a distinct opportunity to help consumers reach their 48 g daily intake target. Ingredients like brown rice, buckwheat, quinoa and other on-trend grains could be considered when formulating new options. In this respect, plant-based meat substitutes could become a vehicle for increasing whole grain consumption.

In order to attract consumers, front-of-pack claims and labelling systems such as the Health Star Rating may be useful in directing consumers to healthier products within the category. Protein content as a claim was used on 60% of products yet a further 17% of products could be using this claim. The same was true for dietary fibre, where 39% of products display the claim yet an additional 19% of products from this review would be eligible to make a claim. It is important to mention that just 4% (*n* = 5) of products would be eligible for a low in sodium claim with products ranging from 58–1200 mg/100 g. The opportunity to utilise quality ingredients, including sources of dietary fibre possibly resulted in the relatively high (mean) HSR (3.6–4.4) for this category of foods, assisting the perception that these products are healthy without exception, yet complicated and extensive ingredient lists and higher levels of sodium may suggest otherwise.

In alignment with recently published guidance from EAT Lancet [13], new dietary guidelines from Canada [16], and revised recommendations from the Australian National Heart Foundation [11], changes in other national guidelines reducing meat may come sooner than consumers realise, and there are strong suggestions that Australians are not ready for such change [55,56]. Rather than totally removing meat from the diet, the pressure will be to reduce the frequency of consumption and serve size [27,56]. Even this requires dietary change and neither sustainability or health approaches are likely to work with those who have strong positive beliefs about meat eating [57]. Plant-based meat substitutes may help disrupt the negativity around reducing meat, and may be an effective foil for both campaigns; however, it is clear that some attention to nutrient composition and equivalence to meat is required from manufacturers to ensure those with the greatest health literacy do not reject plant-based meat due to the detailed nutrition information on the pack. Ethically, it is in the interests of good public health policy to ensure equivalence.

The strengths of this study include its comprehensive focus. To our knowledge, it is the first study to focus exclusively on plant-based products ‘mimicking’ meat products on shelf in Australia (with others such as tofu, tempeh, and falafel excluded), with a comparison made to animal meat products. Also, where HSR was not provided, this was calculated for a more accurate representation of HSR across the category. However, there were some limitations. While all efforts were made to capture the category in its entirety, differences may exist between geographic areas. The reported ingredients and NIP was not validated with independent nutrition analysis and, therefore, some nutrients were unable to be compared. Furthermore, meat burgers were not available via the FSANZ food-composition database.

## 5. Conclusions

Plant protein is on-trend and growing globally, but this overview of plant-based meat substitutes demonstrates sodium is an issue for these products and, importantly, this nutrient is the leading dietary factor in terms of the global burden of disease. Manufactured foods present an opportunity to assist with sustainability issues, and improve health and disease, but some rigor around nutrient targets like those in place in the UK, may be needed for product development, and this may encourage further support and longevity for the category. In comparison to similar animal meat products, plant-based meat substitutes may have the appearance of being a healthful option, but on closer inspection, care and some guidance may need to be provided to consumers about how to construct plant-based diets. Overall, the products lack equivalence with similar meat products, a limitation for vegetarian/vegans and meat consumers alike who may fall short of key nutrients. Balanced messages about not needing to be entirely meat free may be necessary from both a sustainability and nutritional point of view. Overall, the category presents as an opportunity to meet whole grain targets and increase consumption of legumes in a convenient food form with known acceptability among consumers.

## Figures and Tables

**Table 1:** Classification of categories.

Category	Description
Burgers	Meat-free patties, including either ‘burger’, and/or ‘pattie/ patty’ in the product name
Sausages	Features either ‘sausage’, or ‘hot dog’, in the product name
Mince	Features ‘mince’ in the product name
Chicken	Meat-free products appearing to mimic chicken, including “Chick’n Nuggets,” “Nuggets,” “Chicken-Style Strips,” and “Vegan Schnitzel.”
Seafood	Meat-free products appearing to mimic seafood, including “Fish-Free Fingers,” “Battered Prawn-Style Pieces,” and “Tuno.”
Other	Meat-free products falling outside of other categories, including “Vegie Roast,” “Bacon-Style Rashers,” and “Polony.”

**Table 2:** Changes in product numbers and type of plant-based meat substitute product between 2015 and 2019 audits.

Category	2015 Total Products (*n* = 26)	2019 Total Products (*n* = 137)	Increase
Burgers	7	50	614%
Sausages	6	29	383%
Mince	5	10	100%
Chicken	4	24	500%
Seafood	0	9	
Other	4	15	275%
Total	26	137	429%

**Table 3:** Nutrients and Health Star Rating (HSR) per 100 g plant-based burgers, sausages, mince, chicken, seafood, and other plant-based meats (mean ± standard deviation (SD) and median (range)).

Nutrient Criteria.	Burgers (*n* = 50)		Sausages (*n* = 29)		Mince (*n* = 10)		Chicken (*n* = 24)		Seafood (*n* = 9)		Other (*n* = 15)	
	Mean ± SD	Median (Range)	Mean ± SD	Median (Range)	Mean ± SD	Median (Range)	Mean ± SD	Median (Range)	Mean ± SD	Median (Range)	Mean ± SD	Median (Range)
Energy (kJ)	736 ± 194	736 (355–1160)	735 ± 155	713 (458–1103)	574 ± 238	466 (312–950)	830 ± 208	890 (274–1130)	674 ± 320	762 (231–1178)	778 ± 205	805 (405–1180)
Protein (g)	9.6 ± 5.1	7.1 (2.9–20.9)	13.4 ± 6.0	14.2 (2.8–23.0)	13.7 ± 6.0	14.9 (4.6–23.4)	13.4 ± 7.6	11.9 (1.2–36.1)	8.9 ± 4.3	9.4 (0.3–14.0)	14.5 ± 6.8	14.1 (5.7–26.4)
Fat (g)	7.2 ± 4.8	7.0 (0.5–17.7)	7.9 ± 3.8	7.3 (1.0–19.0)	5.4 ± 5.2	3.8 (0.5–15.0)	9.4 ± 3.3	9.1 (2.0–14.3)	7.6 ± 4.5	8.8 (0.5–12.6)	7.9 ± 6.0	7.1 (1.0–23.2)
Saturated fat (g)	1.5 ± 1.6	1.0 (0.2–8.5)	2.4 ± 2.2	2.2 (0.2–9.7)	2.1 ± 3.1	0.5 (0.1–8.0)	1.6 ± 1.4	1.2 (0.5–5.9)	1.4 ± 1.7	0.8 (0.2–5.2)	1.6 ± 2.6	1.0 (0.4–10.7)
Carbohydrate (g)	16.7 ± 7.2	15.9 (3.9–33.0)	11.4 ± 6.2	10.7 (0.5–24.4)	7.9 ± 7.4	5.6 (1.6–27.0)	12.6 ± 5.7	12.1 (2.7–21.5)	15.1 ± 7.8	14.0 (4.4–30.0)	13.0 ± 8.8	13.5 (1.3–26.7)
Sugars (g)	3.4 ± 3.2	2.6 (0.3–15.5)	2.2 ± 1.9	1.7 (0.5–8.7)	1.9 ± 1.5	1.3 (0.3–4.3)	2.1 ± 1.3	2.0 (0.1–3.9)	3.3 ± 2.2	2.6 (1.0–6.8)	3.2 ± 2.1	2.5 (0.8–7.4)
Dietary Fibre (g)	5.3 ± 2.6	5.5 (0.3–11.3)	4.2 ± 1.8	4.3 (1.1–6.7)	5.9 ± 3.4	5.0 (1.2–12.4)	4.7 ± 1.6	4.8 (2.0–7.2)	3.6 ± 1.4	3.4 (1.8–6.3)	4.9 ± 2.4	4.8 (0.4–8.2)
Sodium (mg)	372 ± 173	343 (115–773)	497 ± 136	496 (271–801)	401 ± 310	313 (58–1200)	508 ± 240	488 (220–1196)	447 ± 266	370 (117–900)	568 ± 283	550 (197–1030)
Iron (mg)	3.6 ± 0.8	3.5 (2.8–4.7)	3.4 ± 0.4	3.5 (2.5–3.5)	2.8 ± 1.0	3.5 (1.2–3.5)	4.8 ± 2.2	3.9 (3.5–9.1)	Not Reported		3.2 ± 0.9	3.0 (2.3–4.7)
HSR	4.1 ± 0.6		3.8 ± 0.7		4.4 ± 1.2		3.6 ± 0.5		3.8 ± 0.3		3.6 ± 0.7	

**Table 4:** Nutrients and Health Star Rating (HSR)/100 g (mean and standard deviation) for plant-based and meat burgers, sausages and mince.

Nutrient Criteria	Plant-Based Burger (*n* = 50)	Meat Burger *	*p* Value	Plant-Based Sausages (*n* = 29)	Meat Sausages **	*p* Value	Plant-Based Mince (*n* = 10)	Meat Mince **	*p* Value
Energy (kJ)	736 ± 194	760 ± 257	0.737	735 ± 155	1157 ± 287	0.001	574 ± 238	774 ± 162	0.035
Protein (g)	9.7 ± 2.6	15.4 ± 2.6	<0.001	13.4 ± 6.0	16.0 ± 3.1	0.081	13.7 ± 5.6	25.1 ± 4.0	<0.001
Fat (g)	7.2 ± 4.8	13.7 ± 7.8	0.001	7.9 ± 3.8	22.1 ± 8.4	<0.001	5.4 ± 5.2	9.4 ± 3.6	0.053
Saturated fat (g)	1.5 ± 1.6	6.2 ± 4.1	0.005	2.4 ± 2.1	8.5 ± 1.6	<0.001	2.1 ± 3.1	3.9 ± 1.7	0.108
Carbohydrate (g)	16.7 ± 7.2	5.2 ± 1.9	<0.001	11.4 ± 6.2	3.7 ± 1.5	<0.001	7.9 ± 7.3	0	
Sugars (g)	3.4 ± 3.2	1.3 ± 0.9	0.046	2.2 ± 1.9	0		1.9 ± 1.5	0	
Dietary Fibre (g)	5.3 ± 2.3	NA		4.2 ± 1.8	0.6 ± 0.4	<0.001	5.9 ± 3.4	0	
Sodium (mg)	372 ± 1173	463 ± 119	0.119	497 ± 136	826 ± 142	<0.001	401 ± 310	64 ± 12	0.007
Iron (mg)	3.6 ± 0.8	Not Reported		3.4 ± 0.4	3.6 ± 1.0	0.529	2.8 ± 1.0	2.1 ± 1.1	0.2
HSR	3.9 ± 0.4	2.9 ± 0.9	0.005	3.8 ± 0.6	1.4 ± 0.2	<0.001	4 ± 1.2	4.2 ± 0.3	0.623

Independent samples *t*-test 95% CI; * Data from Coles and Woolworths online (accessed September 2019); ** Data for meat-based mince and sausages from Food Standards Australia New Zealand (FSANZ) Food Composition Database [35].

**Table 5:** Ingredients in plant-based meats contributing to key nutrients.

Nutrient	Ingredient Listed
Protein	Soy protein, pea protein, soy beans, hydrolysed vegetable protein, mycoprotein, almonds
Fat/Saturated Fat	Vegetable oil, canola oil, sunflower oil, sunflower kernels, rice bran oil, coconut oil, flax seed meal, cocoa butter, peanuts
Carbohydrate/Sugars	Potatoes, tapioca, rice flour, sweet potato, corn starch, potato starch, sugar, fructose, apple, tomato paste, wheat flour
Dietary Fibre	Brown rice, lentils, black beans, wheat fibre, chickpeas, quinoa, red lentil, locust bean gum, buckwheat, adzuki bean, split peas, green peas, whole pear millet, soy fibre, bamboo, methylcellulose, mushrooms, mung beans, carrot, pumpkin
Vitamins and Minerals	Iron was added to 33/137 products; (via supplemental iron or fortified flour), vitamin B12 was added to 28/137 products; zinc was added to 25/137 products

**Table 6:** Most frequent nutrition content- and health-related claims on packaging.

Nutrition/Health Claim	Total Products Making Claim (%)
Vegetarian/vegan/plant-based	81% (*n* = 112)
Protein	60% (*n* = 83)
Dietary fibre	39% (*n* = 54)
Non-Genetically-Modified	35% (*n* = 48)
Dairy-free	26% (*n* = 36)
Gluten-free	26% (*n* = 36)
No artificial colours/flavours/preservatives	21% (*n* = 30)
Vitamins	19% (*n* = 26)
Minerals	21% (*n* = 29)
Fat	15% (*n* = 20)

## Appendix A

**Table A1:** Comparison with Healthy Food Partnership proposed reformulation targets.

Food Category	Nutrient	Proposed Target	Plant-based Meat Equivalent (*n* =)	Plant-based Meats Meeting Proposed Targets (%)
**1. Sausages**	Sodium	540 mg/100 g	29	55% (*n* = 16)
	Saturated fat	7 g/100 g	29	96% (*n* = 28)
**2. Crumbed/battered meat/poultry**	Sodium	710 mg/100 g	20	90% (*n* = 18)
**3. Crumbed/battered seafood**	Sodium	270 mg/100 g	5	20% (*n* = 1)
**4. Bacon**	Sodium	1005 mg/100 g	1	0
**5. Ham**	Sodium	1005 mg/100 g	1	100%
**6. Processed meats**	Sodium	720 mg/100 g	1	0
